# The development of an ivermectin-based attractive toxic sugar bait (ATSB) to target *Anopheles arabiensis*

**DOI:** 10.1186/s12936-017-1994-6

**Published:** 2017-08-15

**Authors:** Frank Chelestino Tenywa, Athumani Kambagha, Adam Saddler, Marta Ferreira Maia

**Affiliations:** 1 Ifakara Health Institute, P.O. Box 74, Bagamoyo, Pwani United Republic of Tanzania; 2 Swiss Tropical and Public Health Institute, Socinstrasse 57, 4002 Basel, Switzerland; 3 University of Basel, St. Petersplatz 1, 4002 Basel, Switzerland; 4 Kemri Wellcome Trust Research Programme, CGMRC, PO Box 230-80108, Kilifi, Kenya

**Keywords:** Attractive toxic sugar bait, Recyclable, Mosquitoes, Resting place, Malaria, *Anopheles arabiensis*

## Abstract

**Background:**

An increasing number of countries in sub-Saharan Africa are moving towards malaria-elimination, mostly thanks to successful vector control campaigns. However, elimination has proven challenging, resulting in the persistence of malaria transmission. It is now accepted that in order to eliminate malaria, new complementary vector control approaches must be developed. This study describes the development of a sugar-baited resting place containing a toxic dose of ivermectin for the control of *Anopheles arabiensis*.

**Results:**

Dose response experiments were performed in insectary conditions to determine the LD90 of ivermectin against *An. arabiensis*. Over 95% of *An. arabiensis* were knocked down 48 h post-sugar feeding on 10% sucrose solutions containing 0.01% ivermectin. When investigating different juices as attractants, it was observed that *An. arabiensis* preferred orange, watermelon and commercial guava juice over pawpaw, tomato, mango or banana, but were most likely to feed on simple 10% sugar solution. Using recycled materials, different bait prototypes were tested to determine the best design to maximize sugar feeding. Baits that offered a resting place for the mosquito rather than just a surface to sugar feed were more likely to attract *An. arabiensis* to sugar feed. The optimized prototype was then placed in different locations within a screen-house, colour-coded with different food dyes, containing competing vegetation (*Ricinus communis*) and experimental huts where humans slept under bed nets. Around half of all the released *An. arabiensis* sugar fed on the sugar baits, and approximately 50% of all sugar fed mosquitoes chose the baits close to outdoor vegetation before entering the huts.

**Conclusions:**

Ivermectin is an effective insecticide for use in sugar baits. The design of the sugar bait can influence feeding rates and, therefore, efficacy. Sugar baits that offer a resting surface are more efficient and sugar feeding on the baits is maximized when these are placed close to peri-domestic vegetation. Attractive toxic sugar baited resting places may provide an additional vector control method to complement with existing strategies.

## Background

Malaria across endemic regions of sub-Saharan Africa is declining [1, 2]. The success has been attributed to vector control interventions such as long lasting insecticide-treated bed nets (LLINs) and indoor residual spray (IRS), as well as improved malaria treatment through the adoption of artemisinin-based combination therapy (ACT) as main line of treatment against malaria [3]. Despite the success of these interventions, malaria elimination remains an ambitious target as residual malaria transmission has proven to be a great challenge for malaria control programmes. Residual transmission occurs as result of a combination of human and vector behaviours; mosquitoes can avoid control interventions and feed upon humans when they are not protected while humans are often active late into the night. LLINs and IRS cannot control the mosquitoes that bite outdoors or bite people before they go to sleep under a LLIN. Furthermore insecticide resistance, both metabolic and behavioural, threaten the efficacy of current control tools. New vector control paradigms need to address the shortfalls of current interventions by relying less on pyrethroids insecticides [4], by targeting mosquitoes at different life stages and at different locations other than inside houses [5]. There is undoubtedly a need to think out of the box and develop complementary tools that can narrow the existing gaps [6]. Also, finding a balance between cost and effectiveness in resource poor settings has remained a great concern.

Female mosquitoes feed on sugar mainly from plants in order to obtain energy for survival, flight and fecundity [7]. They quest for sugar before and after obtaining blood meal so as to maintain their fitness throughout their life. Attractive toxic sugar baits (ATSBs) are a new mosquito vector control paradigm that kills both female and male mosquitoes [8–14]. The concept exploits the sugar feeding behaviour of mosquitoes, attracting them to sugar feed on a source containing an insecticidal ingredient. Its mode of action is quite different from current interventions as it targets mosquitoes when they sugar feed rather than host-seeking or resting which are traditionally targeted by LLINs and IRS. In such context, ATSBs may complement with existing vector control interventions. Operationally, the concept can be applied by suspending removable bait stations or spraying on natural sugar sources that surround mosquito breeding sites or human habitats. The intervention is technologically and operationally simple, environmentally safe and cost effective, characterizing its suitability for controlling malaria vectors in low and middle-income countries (LMIC).

Field applications of ATSBs using spinosad and boric acid as insecticides demonstrated the potential effectiveness of ATSBs by decimating *Anopheles sergentii* populations approximately by 95% in Israel [8, 12] and *Anopheles gambiae* sensu lato *(s.l.)* by 90% in Mali [10]. Also >90% of *Anopheles crucians* observed to be stained upon deployment of dyed attractive sugar bait (ASB) in wells and cisterns in Florida [15]. Based on this observation; considerable mosquito mortality could be observed if the baits contained an oral toxicitant. Similar interventions observed to be effective at reducing non-malaria vectors population by approximately 80% in Florida and Israel [12, 15, 16]. These findings underline the importance of ATSB not only on malaria vector control, but also for mosquitoes that are vectors for other diseases.

*Anopheles arabiensis* is currently the most important malaria vector in Tanzania and other parts of Eastern Africa. It shows a high degree of phenotypic plasticity with the ability to adapt its host-seeking and resting behaviour [17]. Although its host-seeking behaviour is well characterized, its sugar feeding behaviour is very poorly documented. Similarly, to host-seeking, sugar feeding is also done selectively. Studies conducted in Mali and Kenya reported feeding preference of *An. gambiae* sensu stricto (*s.s.)* on particular plants, such as *Acacia macrostachya, Acacia albida, Boscia angustifolia, Ziziphus mauritiana* [14], *Parthenium hysterophorus*, *Tecoma stans* plants [18]. The selection of the toxicant to be included in an ATSB has remained a scientific concern as non-target species may be affected and also children may be attracted given the sweet nature of the substrate [19]. To counter these concerns, this study will use the ivermectin as mosquito oral toxicant due to its safety records in humans.

## Methods

This study aimed at developing a mosquito killing bait station that could be made at home using household materials. The ideal prototype was designed after investigating the optimal structure, substrate and deployment location of the bait. A stepwise approach to each design feature was performed using recycled materials, simple household utensils and ingredients that can easily be found in rural sub-Saharan Africa. First, the minimum effective concentration of ivermectin was determined, then different attractive fruit juices were tested, followed by different designs and last the final bait prototype was tested inside a semi-field system (SFS) to determine how effective the baits were when placed in different peri-domestic locations.

### Determining the ivermectin LD90 against *Anopheles arabiensis*

Ivermectin was selected as the mosquitocidal ingredient, given its proven safety record in humans [20]. The endectocide’s mode of action makes it safer to vertebrates including humans as it targets glutamate-gate chloride channel present in invertebrates [20]. The channel does_not exist in vertebrates [21] and the drug has a low affinity for other mammalian ligand-gated chloride channels. Furthermore the drug is unable to cross the blood–brain barrier [21]. In addition, ivermectin is easily available and affordable in rural Eastern Africa, as communities commonly use it to deworm their cattle under the form of injectable Ivomec®. The drug is stable at room temperature and may be stored at 30 °C without losing its efficacy. Reports on ivermectin indicate there is gradual photo-degradation of the chemical when is in animal’s faeces [22]; however little information on whether ivermectin solution undergoes photo-degradation exists. Moreover the drug has a different mode of action to current insecticides highlighting the possibility of circumventing the issue of emerging insecticide resistance. There may be potential for cross-resistance between ivermectin and pyrethroid insecticides though little evidence of this currently exists and further investigation is needed [23, 24].

The minimum dose of Ivermec® needed to kill at least 90% of *An. arabiensis* was determined in the laboratory using dose response experiments inside standard insectary cages (30 × 30 × 30 cm). The mosquitoes used were *An. arabiensis* Ifakara strain, reared at 28 °C, 80% humidity and natural light conditions at the Ifakara Health Institute (IHI) insectaries in Bagamoyo, Tanzania. Larvae were reared on Tetramin® fish flakes, adults were maintained on 10% w/v glucose and blood feed on human blood for colony maintenance. The mosquitoes used in the dose response experiments were blood-naive, 3–6 days old and starved for 6–8 h before experiments. Serial dilutions of ivermectin in 10% w/v sucrose solution were prepared with reference to data collected by Allan [25], who reported that 0.014% of ivermectin in 10% w/v sucrose solution was sufficient to kill 90% of *Anopheles quadrimaculatus*. A food colouring agent (Carmoisin) was added to each dilution at concentration 0.5% v/v in order to easily identify if the mosquito had taken a sugar meal. Injectable Ivomec^®^ was purchased at a “*duka la mifugo*”, local village shop selling veterinary and agricultural products, and used to create serial dilutions of ivermectin. A total of four replicates were performed for each ivermectin concentration after dilution: 0.2, 0.15, 0.1, 0.05, 0.025, 0.01, 0.005, 0.0025, and 0.001%. Forty female *An. arabiensis* were introduced into each cage. Containers with approximately 30 mL capacity were filled up to 2/3 with the test solutions and standard filter paper was rolled up like a tube and fit into the cup in a way that only the bottom part of the paper was in contact with the solution. The test solution then progressed up the filter paper through osmotic pressure thus allowing the mosquito to obtain a sugar meal from its surface. The test solutions were kept in the cages for around 12 h from 8 p.m. to 8 a.m. Mosquito knock down rate was observed at 3, 6, 24 and 48 h. All the mosquitoes that were no longer flying in the cage were removed with a syphon; their abdomens were squeezed onto white filter paper allowing the investigators to determine if the mosquito had sugar fed by visualizing the food dye that had been ingested. After each replicate the cage positions were changed to avoid any bias introduced due to cage positions.

### Selecting the most attractive sugar concoction

In order to determine the most attractive sugar source to *An. arabiensis* to be used in ATSB; experiments were conducted in semi-field conditions during the months of March and April 2015 in Bagamoyo Tanzania. Average temperature was 28 °C, with minimum temperatures of 23 °C and maximum of 31 °C. A total of six cages (120 × 120 × 120 cm) were locally made using a metal frame, and screened with polyethylene net on all panels except for the bottom panel which was made of wood and lined with a light shade of vinyl floor sheet for easy visualization of knocked down mosquitoes. Each cage had a sleeve made of cloth on one of its net panels allowing easy access to the inside of the cage. Sugary concoctions were prepared using 10% sugar solution added to the pulp of the following locally bought fruits: papaya (*Carica papaya)*, banana (*Musa*), tomato (*Solanum lycopersicum)*, mango (*Mangifera indica)*, orange (*Citrus sinensis)* and watermelon (*Citrullus lanatus)*. Guava juice from Azam^®^ was also purchased and tested based on previous studies conducted in Mali that had shown it to be attractive to sugar-seeking *An. gambiae s.l.* [14]. A control solution was also tested using only 10% sugar solution. Sugar baits were made using a used 0.5 L plastic water bottle, cut in half and lined with cotton cloth folded over its outer surface. When the concoctions were added to the bottle the liquid moved to the outer cloth through osmotic pressure creating a surface where mosquitoes could easily sugar feed. The baits were hung in the centre of each experimental cage. For each experimental round different fruit concoctions were compared to 10% w/v sucrose solution. A total of 40 starved female *An. arabiensis* were introduced into each cage. After 24 h mosquitoes were removed and feeding success was recorded by observing the food dye in the mosquitoes’ abdomens. Four replicates were done for each concoction type. This experiment was used to identify the sugary concoction that was most attractive to *An. arabiensis* and so most appropriate in a sugar bait.

### Selecting the prototype design

Different bait/trap prototypes were designed using items commonly found in rural Tanzanian households. The aim was to create a prototype that effectively attracted mosquitoes searching for a sugar meal using basic domestic materials, easily available and with a minimal associated cost. Therefore, preference was given to materials that were commonly reused or thrown in the waste. Discussions were commonly held with locals regarding different materials that could be considered for this purpose. Three different prototypes, denominated A, B and C, were designed using materials such as cloth (“kanga”, “kitenge” and “tetroli”), different sized plastic bottles, string and pieces of sponge from old mattresses. Prototype A was made by cutting a 0.5 L plastic water bottle in half then lining it with cotton cloth folded over its outer surface, prototype B was made by cutting a 12 L bottle (‘*maji ya uhai’)* placing on its bottom a fitting piece of sponge and lined with black cloth; and prototype C was made by cutting 1.5 L bottle into half then the upper part of the cut bottle was seeped into the bottom part, the two parts were fixed with masking tape. The prototype C resembled the “honey trap” with the exception that it did not contain yeast. In order to maintain the CO_2_ production, yeast would need to be constantly added to the trap thus increasing the cost of the prototype making it unattractive to local communities.

Each type of prototype was assigned to one pair of experimental cage and tested using the best performing concoction determined in the previous objective. One prototype with ivermectin and another without ivermectin was placed into each cage in order to determine if mosquito feeding was deterred by ivermectin when using different prototype designs. A total of 40 starved female *An. arabiensis* were introduced into each cage. After 24 h mosquitoes were removed and feeding success was recorded by observing the food dye in the mosquitoes’ abdomens. Four replicates were done for each prototype.

### Selecting the potential deployment location within the peri-domestic area

Experiments to determine the best deployment location of the prototype were conducted in a large screen house (22 × 29 m), also known as a biodome or semi-field system, during the months of May and June 2015, in Bagamoyo, Tanzania. The walls of the biodome are made of netting, which allows airflow, thus maintaining similar climatic conditions as outdoors but in a controlled environment (semi-field conditions). The biodome rests on a concrete slab surrounded by a narrow water trench that prevents ants and other animals from invading it and predating on the mosquitoes released during experiments. The biodome is roofed with polyvinyl sheets and divided into two compartments separated by a 29 × 4 m corridor. Each compartment contains two experimental huts (6.5 × 3.5 × 5.1 m). The experimental huts mimic traditional Tanzanian rural households in terms of size, structure and mosquito exit/entry points (eaves, windows and doors). Mosquito exit traps were fitted to the windows of the experimental huts and netting flaps were attached to the inner side of the eaves in order to funnel mosquitoes into the hut when entering it, but not allowing them to exit through the eaves. The exit traps worked in a similar way where mosquitoes are funnelled into the trap when attempting to exit the hut through the window but cannot return back into the experimental hut. Once a mosquito enters the hut the only way it can exit it is through an exit trap.

**Fig. 1:**
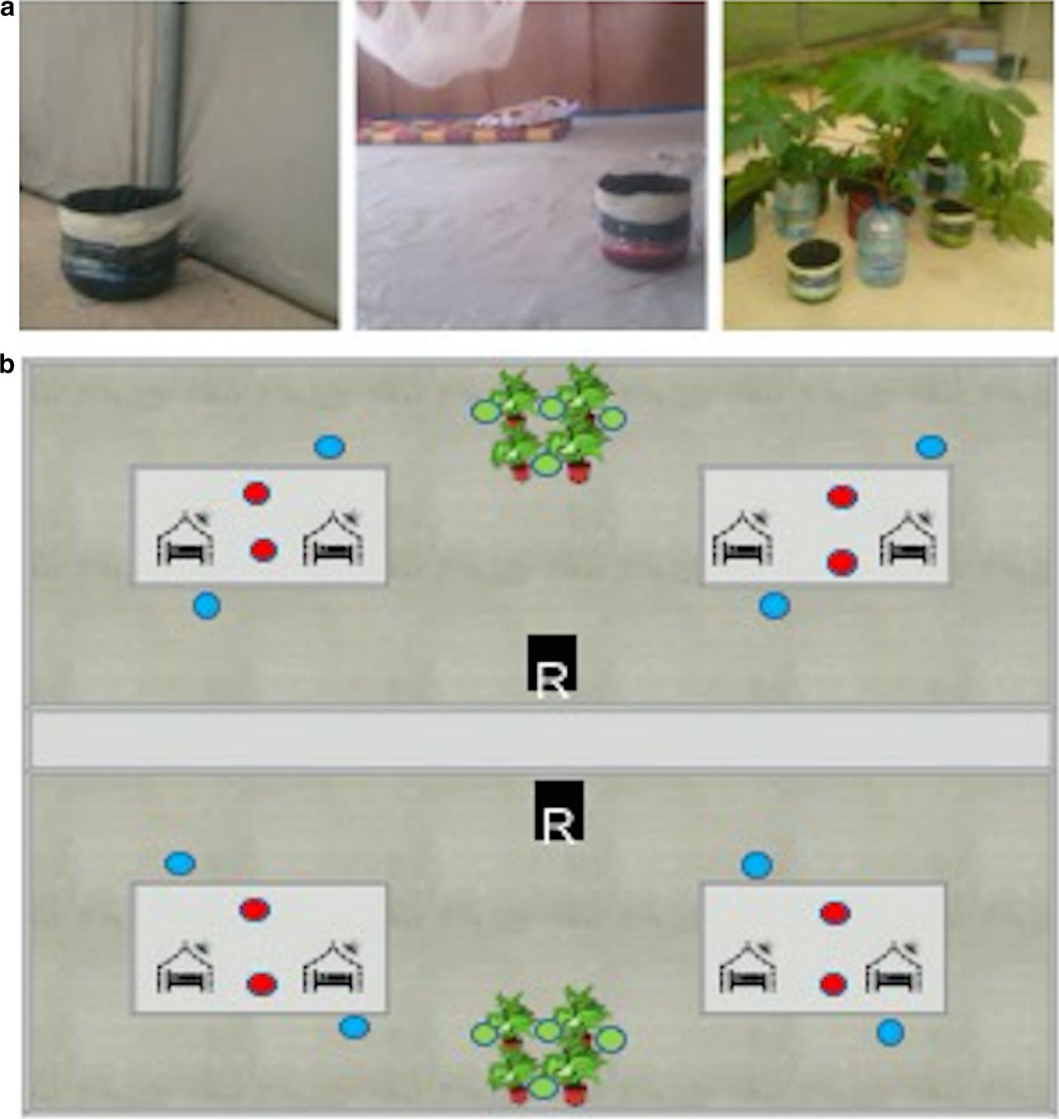
Study design in a semi field system. **a** ASB diployed outdoor around the vegetation, outdoor close to the experimental huts and inside the huts in a biome. **b** Schematic of study design. The biome has two compartments (**a** and **b**); in each compartment two experimental huts are installed. In both compartments; vegetation were potted between the huts. *Green*, *blue* and *red* coded baits were deployed around the plants, close to the huts and inside the huts respectively. Treated and untreated bed nets were installed in huts in compartment **a** and **b** respectively. One hundred and fifty mosquitoes were released in each compartment per night while two sleepers sleeping in each experimental hut

Two mattresses were placed inside each hut and volunteers were asked to sleep in the huts from dusk until dawn. In order to determine the effect of treated and untreated bed nets on mosquito sugar feeding; two huts in one biome’s compartment were given Olyset^®^ LLINs and the other two in another biome’s compartment were given non-treated nets. Four potted *Ricinus communis* plants were placed at the midpoint of each compartment in between both experimental huts. The best performing prototype and sugary concoction determined in previous experiments was used for this experiment. A total of 24 sugar baits were made and deployed at different locations of the biodome. Each location type was assigned a different food dye in order to be able to recognize where the mosquito had fed: (a) eight sugar baits were placed indoors (2 per hut) containing red food colouring; (b) eight sugar baits were placed outdoors directly outside the huts containing blue food dye; and (c) eight sugar baits were placed outside amidst the *R. communis* vegetation containing green food dye (Fig. 1a, b). To maximize outdoor mosquito recapture three artificial resting boxes were randomly placed in outdoor locations. A total of 150 female mosquitoes were released each night in each of the compartments. All dead and alive mosquitoes in each collection site (exit trap, ceilings, resting indoors in baits, resting outdoors in boxes, on plants and outdoors in baits) were separately collected at 07:00. Alive mosquitoes were then knocked down in a freezer and inspected for presence or absence of food colouring in their midguts by squeezing their abdomens onto white filter paper. While inspecting the presence or absence of food dye in the mosquito midgut; observation on whether mosquito had half or fully sugar fed was investigated. A total of 16 nights were conducted, nets and baits were maintained in fixed locations and volunteers were rotated in order to control any bias caused by differences in individual attractiveness to mosquitoes.

### Consideration on the ATSB safety

Into each prototype, one litre of ASB solution was sufficient to make the sponge mattress wet enough for mosquito to feed. In total 10 mL of 1% ivermectin was used to make 0.01% ivermectin concentration needed per prototype. Since this amount of ivermectin contained in one prototype is more than the ivermectin recommended dose for children with 15 kg, it is important to grill the prototype when is applied in the field in order to assure the ATSB trap’s safety. Currently there is inadequate information on the possibility of non-targeted organisms feeding on these traps; however more studies should be conducted to investigate the possible adverse effects of these traps to non-targeted organisms.

### Data analysis

Data analysis was done with STATA version 13 (Stata corp, College Station, TX).

#### Ivermectin LD90 against *Anopheles arabiensis*

A binomial generalized linear model with a logit link was used to compare the cumulative proportion of knocked down mosquitoes for ivermectin concentration to control (10% sucrose solution) at 6, 24, and 48 h. Other sources of variation, such as experimental day, replicate, mosquito age and cage position in the insectary were included also in the model. Odds ratio and 95% confidence intervals were derived from the model.

#### Sugar concoction and prototype design

A binomial generalized linear model with a logit link was used to compare the proportion of sugar fed mosquitoes on each different sugar concoction containing ivermectin compared to control (10% sugar solution). Other sources of variation such as experimental day, replicate, mosquito age and cage position in the insectary were included also in the model. Odds ratio and 95% confidence intervals were derived from the model. The same analysis was done with the data from the different prototype designs using prototype A without the addition of ivermectin as reference.

#### Selecting the deployment location within the peri-domestic area

A logistic regression was done to compare the number of mosquitoes that took a full sugar meal or a half sugar meal between the three possible locations: indoors, outdoors close to the huts and outdoors close to vegetation. The model took into account the day as a random effect; the experimental hut and the net type as fixed effects. Sample size calculations for the ivermectin dose response experiments, selection of the most attractive concoction and selection of the best prototype experiments were carried out in a similar fashion. For each experiment, to be able to detect a minimum of 20% difference in outcome with an alfa of 0.05 and 95% confidence interval; it was calculated that a minimum of forty mosquitoes were needed per arm.

## Results

### Ivermectin LD90 against *Anopheles arabiensis*

**Fig. 2:**
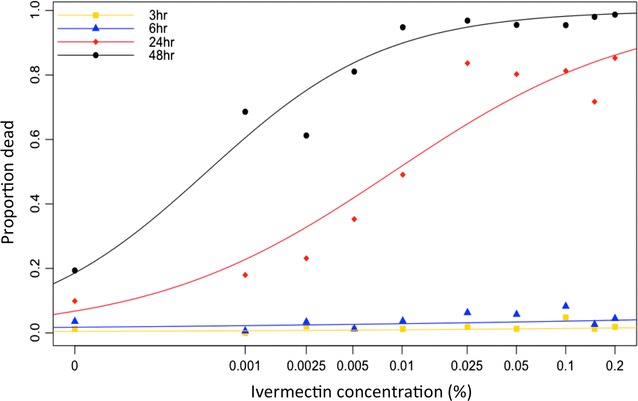
Mean cumulative proportion of *Anopheles arabiensis* knocked down post sugar feeding on different concentrations of ivermectin in 10% sucrose solution for 6, 24 and 48 h

Ivermectin was notably toxic to *An. arabiensis* (Fig. 2). Mosquito knocked-down was evident 24 h after introduction of a sugar meal. Over 80% mortality was observed in a sugar meal containing 0.005% ivermectin after 48 h. Sugar solutions containing 0.01% ivermectin and above; caused approximately 95% mosquito mortality after 48 h (Fig. 2). There was no need to raise the concentration over 0.01% as mortality at this concentration and time observed to be ≥95%.

### Sugar concoction and prototype design

*Anopheles arabiensis* mosquitoes preferred to feed on orange (*Citrus sinensis)* to other fruit juices and just 10% sucrose solution (Table 1). From all the juices tested, orange resulted into the highest number of sugar fed mosquitoes, however it was not significantly more attractive than just sugar solution (OR = 1.02; CI [0.56–1.87]; *P* value = 0.951). The prototypes significantly differed in their ability to attract mosquitoes to sugar feed (Table 2). Prototype B was 3 times more likely to attract *An. arabiensis* than the other prototypes (OR = 3.18; CI [1.63–6.18]; P = 0.001) (Table 2).

**Table 1:** Sugar feeding preference of *Anopheles arabiensis* to different fruit juices containing 0.01% ivermectin compared to 10% sugar solution with 0.01% ivermectin

Concoction	N	n	OR	95% CI	P value
10% sucrose solution	8	304	1	–	–
Banana	8	291	0.33	(0.21–0.50)	0.001***
Papaya	8	296	0.52	(0.29–0.92)	0.026*
Tomato	8	318	0.51	(0.31–0.83)	0.007**
Mango	8	303	0.36	(0.22–0.57)	0.001***
Orange	8	337	1.02	(0.56–1.87)	0.951
Guava	8	315	0.63	(0.40–0.98)	0.042*
Watermelon	8	302	0.70	(0.37–1.35)	0.289

Statistically significant at * p < 0.05, ** p < 0.01, *** p < 0.001

*N* number of replicates, *n* total number of mosquitoes, *OR* odds ratio, and *95% OR-CI* 95% confidence interval or odds ratio

**Table 2:** Odds ratio of *Anopheles arabiensis* fed on three different prototypes containing 10% sugar solution treated with 0.01% ivermectin

Treatment	N	n	OR	95% OR-CI	P value
Prototype A control	4	161	1	–	–
Prototype A + IVM	4	148	1.25	(0.68–2.30)	0.47
Prototype B control	4	167	2.51	(1.45–4.34)	0.001***
Prototype B + IVM	4	134	3.18	(1.63–6.18)	0.001***
Prototype C control	4	150	0.56	(0.23–1.32)	0.183
Prototype C + IVM	4	153	0.35	(0.12–0.99)	0.048*

Statistically significant at * p < 0.05, ** p < 0.01,*** p < 0.001

*N* number of replicates, *n* total number of mosquitoes, *OR* odds ratio, *95% CI-OR* 95% confidence interval of odds ratio, *IVM* ivermectin

### Potential ATSB deployment location within peri-domestic area

Recapture levels were around 57%. 55% of the recapture being caught in the exit traps of the experimental huts. Approximately, 49% of the recaptured *An. arabiensis* had taken a sugar meal. Mosquitoes were more likely to feed on sugar baits that were placed outdoors with 51% of the recaptured fed mosquitoes, fed on baits deployed close to the vegetation (Table 3). Given the design of the experimental huts, mosquitoes once inside could not leave other than through the exit traps, it was observed that mosquitoes sought a sugar meal before being attracted to enter the hut in search of human blood. Mosquitoes that fed outdoors were also more likely to only take half a sugar meal, compared to those feeding indoors which were more likely to engorge in the sugar solution (Table 3). Slightly fewer mosquitoes were caught in the huts with treated bed nets (Olyset nets) compared to those with untreated bed nets (Safi net). However the difference was not statistically significant.

**Table 3:** Total number of mosquitoes that had sugar fed (partially and fully engorged) and their mean proportions

	N	T sugar fed	MPM sugar fed	95% CI-prop fed	T engorged	T partial sugar fed	M prop fully engorged	95% CI prop engorged	P value
Indoors	16	423	0.32	[0.28–0.37]	110	313	0.26	[0.21–0.31]	0.001**
Outdoors near hut	16	226	0.15	[0.1–0.19]	50	176	0.23	[0.17–0.30]	0.001**
Near-by vegetation	16	680	0.51	[0.4–0.55]	108	572	0.16	[0.13–0.19]	0.001***

Statistically significant at * p < 0.05, ** p < 0.01,*** p < 0.001

The mean proportion of sugar-fed mosquitoes, 95% confidence intervals and P value were derived from a logistic regression model analysing differences in proportion of sugar-engorged mosquitoes fed from three different types of deployment sites: indoors, outdoors close to the hut and outdoors close to vegetation

*N* number of replicates, *T sugar fed* total number of recaptured mosquitoes that had taken any type of sugar meal, *MPM sugar fed* mean proportion of sugar fed mosquitoes (excluding unfed), *95% CI prop fed* 95% confidence interval of mean proportion of sugar fed mosquitoes (excluding unfed), *T engorged* total number of recaptured mosquitoes fully engorged with sugar, *T partial sugar fed* total number of recaptured mosquitoes partially sugar fed, *M prop mosq engorged* mean proportion of mosquitoes fully engorged with sugar of the mosquitoes that sugar fed (Prop mosq engorged = T sugar fed/T engorged); *95% CI-prop engorged* 95% confidence interval of mean proportion of mosquitoes fully engorged with sugar

## Discussion

The results from this study conclude that ivermectin has excellent mosquitocidal properties when ingested by *An. arabiensis* in a sugar meal. There is a directly proportional relation between mosquito mortality and ivermectin concentration in sugar solution. Compared to the control; significant mosquito mortality was observed 24 h post introduction of a sugar meal containing 0.005% ivermectin (Fig. 2). 0.005% and 0.01 ivermectin resulted in approximately 80 and 95% mortality of *An. arabiensis,* respectively, within 48 h (Fig. 2). The observed lethal effect is consistent with reports on *Anopheles* sensitivity to ivermectin [26–28]. Also, areas where humans or animals took part in mass drug administration campaigns using ivermectin for treatment against onchocerciasis and other parasitic diseases, documented a decrease in *An. gambiae* populations [29, 30]. This highlights the effectiveness of ivermectin as mosquitocide regardless the route used to deliver it. Furthermore, reports have described that ivermectin when ingested by mosquitoes causes sub-lethal effects by reducing females’ longevity, egg-hatching rate and survival rate of progeny larvae [27, 30–33]. Including ivermectin in sugar bait is an effective alternative to other compounds, such as boric acid which is quite toxic compared to ivermectin and is harder to purchase in local shops in rural Tanzania.

Mosquito feeding selection on sugar sources depends on factors such as visual and olfactory cues stimuli [7, 34]. In this study, all sugar concoctions were made using the same materials differing just in fruit sources. Therefore the difference in attractiveness among the concoctions (Table 1) may be due to the differences in the scent of the fruit. The most attractive fruit baits were not different from the 10% sugar solution. The advantage of this is that the ATSB solution can be made without the need of adding fruit juices as mosquito attractants, which will reduce the cost and may increase community compliance to ATSB uses. Ecological studies have described mosquito sugar feeding behaviour throughout their life [34–38], the findings highlight the potentiality of using the behaviour in designing new vector control interventions such as ATSB.

The development of attractants mimicking the natural odours that attract a mosquito to sugar feed could result in the development of highly effective toxic bait, as these will strongly compete with the natural sugar resources that are available to the mosquito. The design of the sugar bait is significant to its success. In this study the most effective sugar bait (prototype B) (Table 2) attracted mosquitoes to rest rather than only sugar feed. Mosquitoes were attracted to rest on the dark and moist walls of the bucket-shaped bait and after landing they were tempted to easily feed on available sugar meal containing ivermectin.

It was observed that 66% of *An. arabiensis* sought a sugar meal before entering a hut with a human host (Table 3). This meant that despite the presence of a near-by human, which the mosquito could sense through host seeking olfactory cues, it chose to first sugar feed. Likely, the driving factor for this behaviour was the need for energy required for host seeking. This finding concurs with other authors’ reports which have reported that female mosquitoes feed on sugar sources before host-seeking to improve fitness, flight and fecundity [39–41], while male mosquitoes entirely need sugar throughout their life for survival [42, 43]. Considering the amount of sugar meal taken by mosquitoes with respect to bait deployment locations; mosquitoes were most likely to half sugar feed on the ASBs placed outdoors amongst vegetation compared to those placed indoors. This might be explained by the fact that the outdoors half fed mosquitoes needed sugar for energy required just for flight [7], when questing blood meal from the host. Also it could be that mosquitoes when sugar feeding outdoors still intended to take a blood meal from the close host; so did not fully feed sugar in order to maintain enough space in the midgut for blood. The mosquito behaviours we observed in the biodome could have significant implications for the deployment of ATSBs; however these behaviours still need to be studies in wild populations.

On the other hand, the installed mosquito nets showed an impact on mosquito response to the baits inside the huts. Most of the mosquitoes which sugar fed indoor, observed to be fully fed (Table 3). This observation implies that the mosquitoes which directly entered the huts searching for blood meal were met with a host that was protected under a bed net and therefore had to settle for a sugar meal over a blood meal. Availability and accessibility of the meal sources play great role in mosquito feeding choices [44], therefore, inaccessibility of the human host due to bed net protection driven the mosquitoes entered the huts to fully engorge the sugar solution as there was no any other meal source.

## Conclusions

This study describes the invention of a new malaria vector control tool that combines both resting and sugar feeding behaviour of malaria vectors and describes how it can be locally made using recycled materials. This study showed that very small doses of ivermectin in sugar solution can effectively kill more than 90% of *An. arabiensis* that ingest it. Sugar baits were most effective when placed outside among vegetation but are also effective indoors if people are sleeping under a bed net. Potentially using the ATSB-RPs in both locations simultaneously is the most effective alternative to be used in the field. More studies investigating ATSB-RPS in the field are needed to better understand the impact of this intervention on the vector population and on vectorial capacity. In addition, studies should involve both sugar rich and sugar poor environments as competing sugar sources will likely influence the effectiveness of any intervention that wishes to kill mosquitoes exploiting their sugar feeding habits.
